# Gender Transition: Is There a Right to Be Forgotten?

**DOI:** 10.1007/s10728-021-00433-1

**Published:** 2021-05-02

**Authors:** Mónica Correia, Guilhermina Rêgo, Rui Nunes

**Affiliations:** Faculty of Medicine of the University of Porto, Alameda Prof. Hernâni Monteiro, 4200-319 Porto, Portugal

**Keywords:** Ethics, ‘Right to be forgotten’, Gender transition, Health data

## Abstract

The European Union (EU) faced high risks from personal data proliferation to individuals’ privacy. Legislation has emerged that seeks to articulate all interests at stake, balancing the need for data flow from EU countries with protecting personal data: the *General Data Protection Regulation*. One of the mechanisms established by this new law to strengthen the individual’s control over their data is the so-called “right to be forgotten”, the right to obtain from the controller the erasure of records. In gender transition, this right represents a powerful form of control over personal data, especially health data that may reveal a gender with which they do not identify and reject. Therefore, it is pertinent to discern whether the right to have personal data deleted—in particular, health data—is ethically acceptable in gender transition. Towards addressing the ethical dimensions of the right to be forgotten in this case, this study presents relevant concepts, briefly outlines history, ethics and law of records considering the evolution from paper to electronic format, the main aspects of identity construction and gender identity, and explores the relationship between privacy, data protection/information control and identity projection. Also, it discusses in gender transition the relation between “the right to self-determination”, “the right to delete”, and “the right to identity and individuality”. Conclusions on the ethical admissibility of the ‘right to be forgotten’ to control gender-affirming information are presented.

## Introduction

It is undeniable that the European Union (EU)’s adoption of new legislation concerning personal data protection comes from a technological evolution that has generated a massive collection, conservation, and proliferation of data over the past two decades [2, 7, 15, 18, 65, 69, 82]. This new legislation has significant implications in foreign to EU countries (third countries), especially the United States of America (USA) and developed countries [73]. Recently, the EU faced high risks from data proliferation to individuals’ privacy. The use of Facebook profiles by *Cambridge Analytica* during the 2016 USA presidential election campaign to influence undecided voters for the benefit of candidate Donald Trump is an example of this problem [64]. Consequently, new legislation emerged that seeks to articulate all interests at stake, balancing the need for data flow from EU countries with protecting personal data: the *General Data Protection Regulation* (GDPR) [78]. One of the mechanisms established by this new legal regime to strengthen the individual's control over their personal data is the so-called “right to be forgotten”, the right to obtain from the controller the erasure of records. The literature on this new law is still scarce. From the perspective of medical data—a special category of personal data that is particularly sensitive—scientific literature has a gap that should be filed by discussing the ethical issues associated with this right’s exercise. Although patients do not have a consistent right to delete or modify their health records, this traditional position changes due to technological developments and the effects of data connexion. There are already situations where it is considered legitimate to delete health data [21, 56]. For example, in the United Kingdom, “*confidential treatment requests, including end of life plans, where people ask to be allowed to die at home or enter instructions such as “do not resuscitate” *[16], and personal information that is no longer required included in the NHS database (Spine) can be deleted [16].

In gender transition, the right to be forgotten represents a powerful form of control for the owner over personal data, especially health data that may reveal a gender with which they do not identify and reject. Therefore, it is pertinent to discern whether the right to delete personal data—in particular, health data—is ethically acceptable in gender transition.

At this point, it bears emphasising the complexities of transition because it can take the form of legal, social, and medical, or often some combination of two or three. For instance, some trans people use hormones and change their legal sex but do not have any surgeries. Also, some studies address said complexity mainly because there is scientific evidence of the relationship between stigma and health care vulnerability of transgender and gender-nonconforming people [8, 34, 39, 40, 60–62, 72, 84, 86]. Therefore, notwithstanding other motives to modify registries, their legal impact, or other significant branches of the topic, this article’s scope is to discuss the ethical admissibility of the right to be forgotten to control gender-affirming health information. Indeed, regardless of transition-related surgery, as long as gender transition has legal recognition in public registries and critical documents, we aim to provide an ethical assessment of possible claims to have gender-affirming health information deleted.

Towards addressing this issue, we will start by defining relevant concepts. Briefly, outline history, ethics and law of records considering the evolution from paper to electronic format, the main aspects of identity construction and gender identity and expression, and explore the relationship between privacy, data protection/information control and identity projection. Also, we will analyse in gender transition the relation between the rights to “self-determination”, “delete”, and “identity and individuality”. We present conclusions on the ethical admissibility of the right to delete to control gender-affirming health information.

## Definitions

“Genetic data”—“*personal data relating to the inherited or acquired genetic characteristics of a natural person which give unique information about the physiology or the health of that natural person and which result, in particular, from an analysis of a biological sample from the natural person in question*”—Article 4/13 GDPR [78];

“Health data” or “Data concerning health”—“*personal data related to the physical or mental health of a natural person, including the provision of health care services, which reveal information about his or her health status*”—Article 4/15 GDPR [78];

“Right not to know”—right for individuals not to be informed/acknowledged of personal information [23];

“Right to be forgotten”—“*right for individuals to have personal data erased*” [37];

“Sex”—“*A person’s biological status (chromosomal, hormonal, gonadal, and genital) as male or female. An individual's sex at birth (birth-assigned sex) is usually determined based on genital appearance, with those present usually assuming that other components of sex are consistent with the newborn’s genital sex.*” [84]

“Cisgender person”—“*A person who has a gender identity that corresponds with the gender assigned at birth*.” [41]

“Gender”—“*The attitudes, feelings, and behaviours linked to one's biological sex's experience and expression.*” [84]

“Gender identity”—“*refers to each person's deeply felt internal and individual experience of gender, which may or may not correspond with the sex assigned at birth, including the personal sense of the body (which may involve, if freely chosen, modification of bodily appearance or function by medical, surgical or other means) and other expressions of gender, including dress, speech and mannerisms.*” [41, 85]

“Gender expression”—“*The expression of one's gender identity, often through appearance and mode of dress, and also sometimes through behaviour and interests. Gender stereotypes often influence gender expression.*” [84]

“Gender transition” or “Gender affirmation”—“*refers to the social process of being recognised or affirmed in one's gender identity, expression, and/or role. Although gender affirmation can be theorised as an inherently social process, it must necessarily also be conceptualised as multidimensional with at least four core constructs: social (choice of name and pronoun, interpersonal and institutional acknowledgement and recognition), psychological (internal felt sense of self-actualisation, validation of gendered self, internalised transphobia), medical (pubertal blockers, hormones, surgery, other body modification), and legal (legal name change, legal change of gender marker designation). There is no one single path to gender affirmation—no "one size fits all" approach describes how trans people affirm their felt or expressed gender. Some trans individuals pursue social but not medical gender affirmation; some pursue medical but not legal gender affirmation, and so on. Gender affirmation sometimes, but not always, conforms to binary categories of being female or male. Furthermore, gender affirmation does not require linearly following a discrete series of “transition” events—it is conceptualised as an ongoing process throughout the life course.*” [61]

“Transgender and gender-nonconforming people (trans or gender minority)”—“*People who have a gender identity that is different from the gender assigned at birth. This includes people who might or might not undergo gender reassignment, as well as those who prefer or choose to present themselves differently from the expectations of the gender assigned to them at birth.*” [41].

## A Brief Outlook of History, Ethics and Law of Identity Records

Birth is a fact of autonomous relevance, regardless of motherhood and fatherhood. It is essential even if it is impossible to identify the mother and the father, as is the registries of abandoned children. However, in history, this has not always been the case. Indeed, as Andrade [5] points out, in ancient times and most medieval periods of western history, people ruled themselves according to their geographical origin, family lineage, tribal and religious relationships. Personal distinctiveness was diluted in the groups and communities to which they belonged and not according to their characteristics. However, this perspective began to change at the end of the Middle Ages. Indeed, the state-building process that started in the Renaissance required implementing an administrative mechanism capable of individualising. The individuation and differentiation of people were fundamental for the state to oversee law enforcement and tax collection. The solution was to select criteria by which people could be described. Once observed, compiled and recorded on paper by the state, these identification characteristics made it possible to identify individuals and distinguish them from others [5].

On the other hand, according to Durbach [26], the history of registers in the United Kingdom, for example, suggests that, although people’s documentation primarily addressed the needs of the state, this technique was also favourable to some social classes that participated in the process to the extent that their economic interests benefited. Interest in identification registration arose primarily to secure inheritance rights for wealthier social classes. However, the registration of actual events, such as birth and marriage, was equally important for those who did not own property. In the UK, since the sixteenth century, people have understood that the registration of vital information could guarantee their property rights and their rights to state support in times of economic deprivation [26].

Nowadays, registration has become the first step for the state to secure all forms of citizenship, that is, to ensure that they are acknowledged to benefit from the distribution of social state resources, i.e. to ensure that they are protected by society. Registration allows access to widespread personal and economic development tools such as getting a job or a passport. Indeed, besides identity construction, there are other practical reasons for registering one’s identity. For example, people are interested in registering themselves correctly to receive corresponding identity documents necessary for voting or board an aircraft. Undoubtedly, these are some of the reasons for promoting birth registration as a relevant UNICEF activity in the world [77].

Thus, in contemporary society, facts that have an essential impact on relationships with others, such as birth, parentage, marital status, parental responsibilities, nationality, and gender, are recorded in a mandatory manner. Registration allows safeguarding values and principles of certainty and security as to the effects of the people involved. When registered by the state, these data are compulsory, so there is an obligation to register. Thus, registration has a double role: it is an organisational measure of the state; and is regarded as a means of personal affirmation or self-determination, since individualisation through registration facilitates respect for human dignity. Identity registries represent the person’s affirmation as a unique human being, assisting respect for each individual's human dignity. It should also be acknowledged that identity registries serve other functions, such as surveillance and policing. For instance, allegedly, the REAL ID registration system in the United States has anti-terrorism purposes [57].

Furthermore, given the technological revolution of the last two decades, the state has advanced with registering and archiving personal identification data and health and genetic information. The ease of dissemination and connection of data has increased the need for health and genetic data protection, raising complex ethical and legal issues in gender transition, which we will discuss onwards.

## Gender and Identity Construction

Gender identity substantially relates to human dignity since it plays a vital role in identity construction. Although the right to delete may cover registered realities other than gender, namely, challenges to paternity or maternity established at birth or adoption, gender identity—a personality-related fundamental value—represents an independent scope for ethical analysis given its complexity.

Human beings are usually divided in a binary way, either female or male, based on biological information commonly exemplified and recorded at birth [27]. Usually, people are registered by the state based on the biological information voluntarily provided or shared by the parents or by someone on their behalf. However, there is a difference between registration and other forms of data collection since data can be gathered by other means. Indeed, the observation of facts (medical records, for instance) or a transaction between its holder and someone else (such as data uploaded in a website visited) can reveal a profile [81]. These are the so-called observed data. Additionally, there are derived or inferred data that, through the combination of observation and analysis of voluntary data and derivatives, allow one to deduce trends and make behavioural and personality profiles [49], as is the case of human research.

A significant number of countries continue to address gender through a dualistic classification of biological sex. A pathology-based conception of trans and nonbinary identities still exists, which justifies studies to find the best solutions for altered conditions in genital appearance detected at birth. Thus, the pathology-based literature on this subject addresses the advantages and disadvantages of early surgery. Thomas [75] found that establishing gender after transition-related surgery during childhood is generally an “*imperfect exercise*” since the documented results revealed insufficient long-term functional and psychological effects of an early approach. Therefore, this perspective suggests the maintenance of the “*male genital phenotype, visibly not corrected in girls*, *which, however, is inadmissible for most parents.*” [75]. Also, on this issue, Fallat et al. [29] argue that this condition should not be hidden from children, as this would likely create a disturbance at an age when “*sexual identity and peer-group identification is important.*” [29]. We agree with these authors that we should consider both the child’s and future adults’ needs. In this sense, clinical information, especially that related to the child’s body, should not be “forgotten” or deleted.

At this point, it should be acknowledged that the pathology-based approach has been subject to substantial critique because the establishment of gender does not depend on physical characteristics but identity. Gender expression is another concept related to gender identity, and a person’s gender expression may, or may not, follow their gender identity [84]. For example, some trans individuals who have not transitioned may hold an internal gender identity that they do not externalise via gender expression. Also, a large body of writing, including Articles 5 and 10 of CEDAW—The Convention on the Elimination of All Forms of Discrimination against Women [55],—seeks to dismantle the idea of stereotyped sex or gender roles [84].

The comprehensive notion of gender nowadays still encompasses difficulties in choosing sex for registration under different conditions of the morphological sex of the newborn. There is a risk of being arbitrary, and the registered individual may link to a gender with which they might not identify. Besides, problems to transition it later might happen, according to the jurisdictions of the various countries. This difficulty has led to the suggestion that birth registries should admit a third approach, such as a simple procedure for subsequent alteration, or that reference to sex should be omitted. The United Kingdom discussed this idea on a similar issue in 1947. Attempts to allow birth certificate without the father’s name had limited success but were made as a public health measure and in light of the high mortality rates of out of wedlock children [26]. Recently, critiques of the gender binary view are gaining momentum as a growing number of countries—Belgium, Germany, Greece, Iceland, Malta, The Netherlands—now recognise nonbinary genders in official registries or use no gender markers at all in their official documentation [28].

Still, in some European countries (e.g., Denmark, Ireland, Norway and Portugal), although gender markers are in use, it is unnecessary to undergo gender-affirming surgery for gender transition legal recognition. Gender identity is based on self-determination [41, 53], so people have the right to maintain primary and secondary sexual characteristics even if they chose to transition their gender. The right to self-determine one’s gender identity and gender expression is ensured, namely, through the free development of one’s personality according to identity and gender expression. Denmark evolved to adopt a self-determination policy, but before 2014, it was only after forced sterilisation that a different gender could be legally recognised [10, 66]. The state could change the last digit of the identification document number (even for female; odd for male), which reflected an outdated conceptualisation of gender based on a dualistic understanding of biological sex [63].

## Privacy, Data Protection and Identity Projection

The concept of privacy is not consensual, but we believe it is an expression of human dignity. We will try to demonstrate this point by confronting arguments from relevant literature. Whitman [83], for example, considers privacy very difficult to define because of cultural differences between American and European societies, as so, he argues there is no universal concept of privacy. This author also claims that culture and the law shape the sense of privacy of individuals. Likewise, the foundations of differences in values that the law embodies derive from old social and political dissimilarities. Thus, according to this author, two conceptions of privacy exist, sustained by two main sets of values: in Europe, privacy is an aspect of personal dignity, understood as the right to image, name, reputation, threatened mainly by the media; in the USA privacy is an aspect of freedom, endangered mainly by the government. From this perspective, he claims that it is defenceless to consider human dignity as a universal value, equally felt by any society, regardless of its culture and history.

Despite the outstanding writing style Whitman [83] uses to develop his central argument, the problem with this view is that it reduces the value of human dignity to a matter of honour/image. Human dignity is more profound in our judgment, as it represents an abstract capacity and potential for self-determination, regardless of the ability or concrete will for it. This ability may not even exist, as it necessarily happens when one recognises the human person’s dignity with a mental disability. Thus, human dignity relies upon considering the human being as an end in itself, never a means, and this perspective goes far beyond image or honour. From this angle, human dignity is imposed on the individual himself and considered a universal value. Freedom is then a condition of dignity. However, for freedom to exist, there is another condition: privacy. Therefore, privacy is a condition of human dignity. Indeed, as noted by Ursin [80], “*For don’t we assume that there must be a right not to be watched in certain situations and that we should be able to control other peoples’ access to personal information to some degree? The idea that privacy entails being in control of personal information about oneself again links privacy to autonomy, but in this perspective, autonomy is a precondition of dignity.*”

However, in an era of perpetual technological evolution, we are continually facing new instruments, such as combining the physical domain with the virtual one, the so-called “Internet of Things” (IoT).

Furthermore, the means of collecting, preserving and analysing personal information has increased exponentially [81], since the traditional information registers have added new standards, including robotics associated with artificial intelligence, the use of which allows the control of individuals through the establishment of behavioural patterns. Therefore, the associated ethical issues have grown from concerning a right to privacy and confidentiality (privacy *strictu *sensu) to protect the facts themselves, which are primarily innocuous but, once interconnected, convey the individual’s essence without himself knowing it [22]. Technology is why we have gone from protecting intimacy and privacy to protect personal data, a fundamental human right.

At this point, it bears emphasising that ethics and law view rights differently, although they are correlated. Ethics corresponds to a reflection by the society of a mandatory directive’s choice for its members. That is, it concerns an axiological selection of the behaviours desired by a particular human group. The human being is allowed a wide variety of options in the sphere of personal decision, which we call self-determination. However, individual choices result from internal traits, the environment, and interactions with society, resulting in subjective opinions. Thus, it is necessary to find a minimum neutral standard accepted by the conscience of any individual. These standards of conduct taken by any human being, regardless of their ideological or religious principles, constitute the values ​​that shape ethics. The law, in turn, assesses human conduct being permeable to the facts, as it values them in the construction of its norms. However, the law often brings rules and principles impregnated with ethical values, recognised by the political system as essential to its formation. In this way, legal regimes have moral foundations, such as the dignity of the human person.

Notwithstanding, the right to data protection guarantees an individual the right to dispose of all data relating to his personality, health, personal life, political or religious conviction, race, for example. Thus, it serves to sustain privacy protection in a world where collecting, storing, and intersecting large amounts of data is possible [38, 42, 50, 74]. Under these circumstances, the significance of facts and information previously considered irrelevant increases. Modern technology has created a “remembering-by-default” environment where personal data is recorded and stored indefinitely and shared quickly, resulting in the right to privacy violated, identity itself endangered [81]. Most individuals have many aspects of their identity that they hide from others and this circumstance is not necessarily circumscribed to the fear of embarrassment. They may not even cause that effect at all but, rather, might be elements of their personality that they do not wish to divulge in a given context. For example, someone who likes to sing sacred music or practice boxing may not want to reveal this fact in his/her work environment. The fact itself may not be embarrassing, but the data subject has the right to protect it regardless. They are only exercising the right to be different according to the circumstance, the interlocutor, the moment, the goal and their history. This choice describes the freedom of projection of the self, according to the data holder’s free will, moreover, when those facts have the potential of creating a profile.

## The Right to Be Forgotten and Trans Identity Information

Representing a more robust form of personal data protection, the right to be forgotten has recently emerged. The literature on this subject approaches this new law and its ethical foundation from managerial, legal, and societal perspectives [1, 2, 9, 15, 17, 18, 31, 33, 52, 67, 69, 70]. Regardless of the disciplinary perspective from which the subject is approached, it is stressed the new paradigm created by technology—eradicating one of human memory’s functions—the ability to forget [14, 46, 71]. In general, the “right to be forgotten” is valuable for dealing with outdated, useless or decontextualised information [35, 49]. For example, a transgender person who willingly has gender-affirming surgery obtains gender transition recognition in their official documents (as is the case in several European countries). They may wish to delete the confirmatory clinical record of performing, for instance, trans masculine chest/top surgery, which unequivocally relates them with a gender they do not want to project into society.

As enshrined in European culture and legislation, the right to be forgotten raises interesting ethical questions because it does not limit deleting data of any kind. Patients appear to have the right to exclude their health information if the data are no longer required for reference by the purpose for which it was collected or processed, or even if consent is withdrawn. Besides, it seems this right exists when the data subject opposes processing, and there are no legitimate grounds for rejecting this request; also, when the data has been processed unlawfully—all these reasons following Article 17 of the GDPR [78]. The law has no limits regarding genetic and health data. In this context, any data that can unveil a “self” that the holder does not want to project to society could be subject to the right to be forgotten. Indeed, from a particular perspective, this right, rather than privacy protection, protects identity and self-determination because the right to privacy only deals with protecting private information to prevent it from being divulged in the public sphere. In contrast, the right to identity relates to data transmission into the public sphere: the correct projection and representation. The right to delete associated with the right to personal identity allows reaching information that has already been released to the public [6].

In another approach, the right to identity and individuality is considered a fundamental human right. It may take varied ethical contours, namely, the subjects’ right to be informed about their biological ancestry. Amzat and Grandi [3] point out that individuals are characterised, among other attributes, by “historicity”. This perspective also contemplates the right not to be informed of one’s genetic heritage. That is to say, denying the knowledge of information that concerns the individual: the so-called “right not to know”, resulting from informational self-determination [20], considered the ethical and legal ancestor of data protection [36]. It could be argued that the right not to know and the right to be forgotten have a shared perspective. Both rights promote autonomy and control [4, 49].

Nonetheless, if data exist, even if private and only accessible under limited circumstances, the importance of not knowing can be compromised by unwanted revelations of information [4]. Given that information is registered, regardless of being under seal, the circumstance is different [54]. The right not to know is not assured following the availability of information. From an identity perspective, we argue that the right to be forgotten offers more advanced protection to autonomy than the right to privacy.

In this way, some arguments contribute to the admissibility of the right to forget or delete health and genetic data related to gender transition: those we have already mentioned and the ideas that support transgender rights. As emphasised by Powell et al. [58], these rights are usually based on the circumstance’s congenital nature. That is, they state that gender identity is unalterable and insusceptible of choice. However, human rights-based arguments provide a more challenging foundation for transgender rights. Following this line of thought, we say that human rights give more substantial support for the right to erase gender affirming-related data. Indeed, trans people rights derive from human rights, that is, fundamental rights belonging to all people. People with cisgender or transgender identities are equal in human dignity [43]. According to the *Universal Declaration of Human Rights* [79], “*All human beings are born free and equal in dignity and rights*”.

Nonetheless, when discussing transgender rights as human rights, it is crucial to stress the proportionality principle that is a cornerstone concerning the international human rights system. Indeed, the principle of proportionality consists in assessing the suitability, necessity and balance of state intervention in a specific fundamental right. It is based on moderation and justice logic that should apply to any state intervention in individuals’ rights, even if the restrictive act’s purpose is to avoid harm to another individual right [44].

The societies that developed after World War-II under the guidance of the abovementioned international instrument sought to translate, through its principles, a plural understanding, encompassing individuals with diverse ethical expectations and different visions of the world. Pluralism of thinking in modern societies leads political systems to respect freedom. That is, citizens have the right to live their lives in the way they understand, being limited only by others’ rights. In this sense, public authorities should only intervene in their citizens’ free choices to avoid harming other individuals [24]. This idea can be found, for instance, in John Stuart Mill’s so-called harm principle, which he introduces by stating: “*The only purpose for which power can be rightfully exercised over any member of a civilised community, against his will, is to prevent harm to others. His own good, either physical or moral, is not a sufficient warrant.*” [48]. In other words, society can interfere in any individual’s freedom only to avoid harm to others, but it cannot do so because it is for that person’s good. Thus, it is natural to suppose that Mill’s defence of individual liberty is founded on utilitarianism [47]. In Mill’s utilitarianism, the right action is to maximise good and utility. The good is, in general, pleasure. Thus, utilitarianism is the ethics of ordinary happiness. Actions are right if they tend to support the greatest global happiness and wrong if not. Individual sacrifice is useless if it does not increase the total amount of happiness. Indeed, the claims of individuals lose importance for the benefit of all [47]

Similarly, as long as the person does not cause harm to others, his freedom must be guaranteed. This idea is accurate even in the case where such interference could produce significant overall gains in happiness.

Notwithstanding the philosophical literature that builds upon Mill’s harm principle [51, 68, 76], some criticise his arguments. It is one of the most debated political philosophy points, e.g. in Rawls’ work [30]. However, despite the apparent tension between his utilitarianism and liberalism, Mill clarifies that his arguments for individual liberty are, in the long run, dependent on the principle of utility [87]. We stress that, in utilitarianism, Mill seems to regard the principle of liberty as a moral rule. He adheres to the notion that individual liberty protection would ultimately maximise general happiness in a civilised society. Because of this, he stresses the liberty principle’s adoption, whose purpose is to prevent each person’s freedom from being excessively repressed by society. Mill believes that the cultivation of individuality is indispensable to the human being as a progressive being. Mill argues that we need freedom so that we can develop our individuality. It seems that Mill’s arguments for the principle of liberty are sustained in his utilitarianism. In this sense, a more significant sum of happiness/utility can be a reality in which the exercise of authority occurs without constraint and would tend to maximise utility. Therefore, Mill’s utilitarianism would protect individuals’ vital interests as minimum conditions for promoting happiness, disapproving of well-being through the limitation of individual liberty. In this perspective, Mill would remain a utilitarian, but his defence of liberty would be compatible with his utilitarianism. Mill sophisticated utilitarianism, making it a theory that better accommodates the legitimate concerns of those who seek to solve problems related to well-being, happiness, rights and justice [47, 48, 87].

In Judaeo-Christian culture, a similar moral imperative stems from the Bible: “*Thou shalt love thy neighbour as thyself*” [11]. In secular terms, this principle reflects a fundamental ethical imperative that requires acting in all circumstances with the responsibility not to affect others’ autonomy and, consequently, of oneself.

At this point, suppose we consider the right to be forgotten as a means to operationalise data protection and privacy, both conditions of identity. In this context, it makes sense to evaluate this legal mechanism for the entire exercise of identity in matters of gender transition. Hence, it is necessary to discuss the limits of the right to be forgotten regarding gender transition information, considering the principle of liberty and the proportionality principle as a foundation in human rights law. Based on these arguments, we argue that the right to be forgotten may raise ethical and legal difficulties in the following circumstances:

- The right to erase health and genetic information related to gender transition makes tracing genetic diseases impossible, especially relevant when dealing with their biological offspring. Thus, direct biological relatives should have access to a collected genetic sample as long as necessary to understand their genetic status better. This difficulty is not potential but of actual relevance because a line of thought gaining momentum is that transgender people should have reproductive rights. The truth is that transgender people may undergo fertility preservation gametes, usually before gender-affirming treatment. Therefore, some scientific literature calls for legal recognition of gender having in mind self-determination. It is stated that the relinquishment of reproductive capacity is no longer a prerequisite for gender transition, recognising that transgender people desire to be parents and are not intrinsically wrong parents merely due to their non-normative gender identities expressions. This perspective is based on equality and human dignity [13, 32, 45, 59].
- Researchers might have to rely on medical records to study how health issues manifest differently based on biological sex at birth [43]. Consequently, the right to delete gender-affirming associated information could be the starting point for exposing certain health research types.
- The right to forget gender-affirming health information could harm selection in terms of high-performance sports. It is not the purpose of this analysis to discuss the binary division in high-performance sports. However, it is well known that hormone levels differ by gender and that this influences sports performance. Although we agree with Lau [43] that basing the selection of athletes on gender markers in identification documents is disproportioned – because it may violate the rights of transgender athletes in terms of the equality principle – the same cannot be said about their health information. To determine if the hormonal level of transgender athletes is equal to that of cisgender athletes is necessary for equity. Thus, deleting this health information would be disproportionate for this purpose.
- The right to be forgotten regarding gender-affirming information might generate ethical conflicts with other circumstances, such as marriage and adoption, whose impediments (depending on the jurisdiction) might be violated by a gender transition. For example, at a marriage, a member of the couple transitions their gender. Different implications might occur as this relationship turns into a same-sex marriage. Depending on the jurisdiction, adoption, and the use of medically-assisted reproduction techniques might be restricted. In contrast, regarding the latter, suppose it is not restricted. The procedure might be endangered if medical history data have been deleted. As abovementioned, the discussion of recognising reproductive rights to transgender people is a matter of equality and human dignity.
- Also, in case of repentance: changing ideas is also an exercise of autonomy, which is why deleting gender-affirming health data can harm the owner, preventing them from exercising the most varied rights if data recovery is not possible;
- The right to be forgotten so far as gender transition is concerned might create the inability to trace the previous identity, and so criminal responsibilities could be avoided, as it would allow identification forgery; moreover, civil responsibilities could be jeopardised, as it might cause contractual and inheritance problems, for example. In this regard, Lau [43], which defends gender recognition as a human right, acknowledges that past-gender information registries are the proportioned measure to prevent fraud and protect public safety. Instead of relying on ID gender markers, governments could legitimately access previous IDs and link them to current ones during background checks on criminal records [43]. Thus, we argue the right to delete in the abovementioned cases would not be proportionate regarding necessary information. The same argument is valid when gender-affirming associated information supports public faith regarding facts that rely on documentary evidence with a link to identity (e.g., certifying academic qualifications or issuing a passport).

Consequently, although the right to be forgotten is in line with the right to self-determination, there are ethical and legal doubts to ponder. Indeed, we presented sound arguments to support the non-deletion of health data. Still, there are already practical solutions to better collect health data from trans people and respect their privacy. Solutions are suggested by Deutsch and Buchholz [25] to improve their health condition, as there is evidence of their vulnerability. Moreover, Cahill and Makadon [19] recommend recording these situations because these people’s marginalisation is often subjected to unequal health care. Therefore, erasing gender-affirming data can contribute to their health exposure.

## Conclusion

Human beings are allowed a wide variety of personal choices influenced by their environment, culture, and psychology. This freedom in the sphere of individual decision-making—or self-determination–is the basis of self-fulfilment. On the one hand, recognising gender identity as a fundamental human right—in the sense of full development of an individual’s personality through self-determination and self-realisation—is accepted as an integral part of respect for human dignity. In contrast, forgetting gender-affirming health data should be regulated at the international level to protect the human person’s inalienable rights and future generations.

Gender identity is intrinsically linked to human existence because it is present from birth, developing at the same time as a personality following the outer and inner worlds of the individual. So naturally, in any debate, there will always be different and complex cultural sensibilities influenced by the environment in which the individuals are inserted, whether it is public opinion influenced by the media and social networks, by political and economic interests, or religious beliefs. In this sense, we agree with Rich and Ashby [63] assertion that bioethics’ role is to promote thoughtful analysis that applies its principles, as Beauchamp and Childress framed [12] towards human beings and their fundamental rights. Autonomy, beneficence and justice shall be the starting point of any regulatory action. Thus, bioethics, by analysing values in a neutral way and with no preconceived notions, seems to be the most acceptable way to promote gender identity issues while fully respecting human dignity.

After examining some inter-related and conflicting issues regarding the right to delete personal data, specifically medical and genetic records, in the context of gender transition, we conclude that the admissibility of this right should be limited and regulated. However, sound arguments accepted by fair-minded people supported by the principle of liberty and the proportionality principle should be the basis of such a decision.

We argue that the right to delete should be restricted to data that do not risk the issues developed in this discussion. Indeed, given that it is impossible to forget by complete deletion, but only to hinder access, this problem’s total solution may lie in technology. It is required to invest in technology that guarantees the maintenance of the information necessary to comply with the restrictions indicated. Nevertheless, the fundamental question is the ethics that must be endorsed in the regulation of this matter. As so, data that affect any of the values referred in the hypotheses previously discussed, i.e., those which might affect the right to self-determination of other individuals, whether present or future, cannot be subject to oblivion, not even to definitive obstruction of technological access. Deleting health and genetic data can undoubtedly undermine the rights of direct biological relatives as well as generations to come, so we argue that erasing them might be considered ethically unacceptable. Gender identity is a personality-related fundamental value but should not prevail without considering other values because it should not be regarded as absolute.

Regardless of the future of information about the past, it urges a discussion about specific features associated with the right to delete health and genetic data in gender transition from an ethical perspective. It is an issue that deserves broad and extensive exploration, which, along with other contributions, warrants bioethicists’ prompt attention.
